# Development of a four-axis moving phantom for patient-specific QA of surrogate signal-based tracking IMRT

**DOI:** 10.1118/1.4966130

**Published:** 2016-11-07

**Authors:** Nobutaka Mukumoto, Mitsuhiro Nakamura, Masahiro Yamada, Kunio Takahashi, Mami Akimoto, Yuki Miyabe, Kenji Yokota, Shuji Kaneko, Akira Nakamura, Satoshi Itasaka, Yukinori Matsuo, Takashi Mizowaki, Masaki Kokubo, Masahiro Hiraoka

**Affiliations:** Department of Radiation Oncology and Image-applied Therapy, Graduate School of Medicine, Kyoto University, Kyoto 606-8507, Japan; Mitsubishi Heavy Industries, Ltd., Hiroshima 733-8553, Japan; Department of Radiation Oncology and Image-applied Therapy, Graduate School of Medicine, Kyoto University, Kyoto 606-8507, Japan; Department of Radiology, Kurashiki Central Hospital, Kurashiki 710-8602, Japan; Department of Radiation Oncology and Image-applied Therapy, Graduate School of Medicine, Kyoto University, Kyoto 606-8507, Japan; Department of Radiation Oncology, Kobe City Medical Center General Hospital, Kobe 650-0047, Japan and Division of Radiation Oncology, Institute of Biomedical Research and Innovation, Kobe 650-0047, Japan; Department of Radiation Oncology and Image-applied Therapy, Graduate School of Medicine, Kyoto University, Kyoto 606-8507, Japan

**Keywords:** four-axis moving phantom, motion management, tracking, IMRT, patient-specific quality assurance

## Abstract

**Purpose:**

The purposes of this study were two-fold: first, to develop a four-axis moving phantom for patient-specific quality assurance (QA) in surrogate signal-based dynamic tumor-tracking intensity-modulated radiotherapy (DTT-IMRT), and second, to evaluate the accuracy of the moving phantom and perform patient-specific dosimetric QA of the surrogate signal-based DTT-IMRT.

**Methods:**

The four-axis moving phantom comprised three orthogonal linear actuators for target motion and a fourth one for surrogate motion. The positional accuracy was verified using four laser displacement gauges under static conditions (±40 mm displacements along each axis) and moving conditions [eight regular sinusoidal and fourth-power-of-sinusoidal patterns with peak-to-peak motion ranges (*H*) of 10–80 mm and a breathing period (*T*) of 4 s, and three irregular respiratory patterns with *H* of 1.4–2.5 mm in the left–right, 7.7–11.6 mm in the superior-inferior, and 3.1–4.2 mm in the anterior–posterior directions for the target motion, and 4.8–14.5 mm in the anterior–posterior direction for the surrogate motion, and *T* of 3.9–4.9 s]. Furthermore, perpendicularity, defined as the vector angle between any two axes, was measured using an optical measurement system. The reproducibility of the uncertainties in DTT-IMRT was then evaluated. Respiratory motions from 20 patients acquired in advance were reproduced and compared three-dimensionally with the originals. Furthermore, patient-specific dosimetric QAs of DTT-IMRT were performed for ten pancreatic cancer patients. The doses delivered to Gafchromic films under tracking and moving conditions were compared with those delivered under static conditions without dose normalization.

**Results:**

Positional errors of the moving phantom under static and moving conditions were within 0.05 mm. The perpendicularity of the moving phantom was within 0.2° of 90°. The differences in prediction errors between the original and reproduced respiratory motions were −0.1 ± 0.1 mm for the lateral direction, −0.1 ± 0.2 mm for the superior-inferior direction, and −0.1 ± 0.1 mm for the anterior–posterior direction. The dosimetric accuracy showed significant improvements, of 92.9% ± 4.0% with tracking versus 69.8% ± 7.4% without tracking, in the passing rates of *γ* with the criterion of 3%/1 mm (*p* < 0.001). Although the dosimetric accuracy of IMRT without tracking showed a significant negative correlation with the 3D motion range of the target (*r* = − 0.59, *p* < 0.05), there was no significant correlation for DTT-IMRT (*r* = 0.03, *p* = 0.464).

**Conclusions:**

The developed four-axis moving phantom had sufficient accuracy to reproduce patient respiratory motions, allowing patient-specific QA of the surrogate signal-based DTT-IMRT under realistic conditions. Although IMRT without tracking decreased the dosimetric accuracy as the target motion increased, the DTT-IMRT achieved high dosimetric accuracy.

## 1. INTRODUCTION

Intensity-modulated radiotherapy (IMRT) has been used in clinical practice for a variety of tumor types and anatomical locations.^1^ However, when treating tumors, particularly those located in the thoracic and abdominal regions, respiratory-induced tumor motion leads to uncertainty during beam delivery.^2^ Compared with three-dimensional (3D) conformal radiotherapy, a complex interplay occurs between the motion of the multileaf collimator (MLC) and that of the tumor in IMRT, leading to underdosage or overdosage in portions of the target volume and/or surrounding tissues.^2–6^

Several respiratory motion management techniques, including forced shallow breathing, breath-holding, respiratory gating, and dynamic tumor tracking (DTT), have been proposed to reduce the uncertainty caused by respiratory motion,^2^ thereby reducing the probability of normal tissue complications.^7,8^ Of these, recent interest has focused on the DTT technique, which is able to dynamically reposition the radiation beam or the robotic couch in accordance with the position of the target.^9–24^ DTT decreases internal uncertainties due to target motion, without the need for a prolonged treatment time or the burden of shallow breathing or breath-holding for patients. There are two approaches to achieving DTT: direct and indirect tracking methods.^2,9^ To track a moving target, both methods need to predict the future target position to compensate for system lag. The direct method tracks the internal target position itself, whereas the indirect method requires an external surrogate signal to localize the target position according to the correlation between the motions of the internal target and the external surrogate. There are also several approaches to localize the internal target position: dual fluoroscopic imaging systems,^11–16,18,19^ an electromagnetic transponder,^17^ four-dimensional (4D) computed tomography (CT),^20–22^ combined 4DCT and daily cone-beam CT (CBCT) projection images,^23^ and 4DCBCT.^24^

The hybrid beam delivery technique, which combines IMRT with a respiratory management technique, such as breath-holding and gating, has been used clinically.^25,26^ The Vero4DRT (Mitsubishi Heavy Industries, Ltd., Hiroshima, Japan; and Brainlab AG, Feldkirchen, Germany) uses a novel irradiation technique that combines surrogate signal-based DTT with IMRT (DTT-IMRT) using an orthogonal gimbaled MV x-ray head that can swing the radiation field by up to ±41.9 mm, with a maximum speed of 152 mm/s in each direction in the isocenter plane. However, IMRT-related and DTT-related uncertainties are nevertheless included in the dose calculation and treatment of DTT-IMRT. Examples of IMRT-related uncertainties are the leakage profile, round leaf and tongue-and-groove effects, and MLC mechanical errors, which can be confirmed via dosimetric quality assurance (QA) by delivering the treatment plan to a phantom. Additionally, a large source of dosimetric error is the interplay effect between the MLC and the motion of the target.^2–6^ Generally, these interplay effects can be minimized by using respiratory motion management techniques. DTT-related uncertainties, including the prediction error and tracking response error, are also known to cause dosimetric errors depending on the patient’s respiration.^11^ To evaluate these uncertainties, a moving phantom that can realistically reproduce the patient’s respiration, as well as mount a dosimetric QA phantom, is needed.

In this study, we developed a novel four-axis moving phantom that can reproduce patient 3D tumor and one-dimensional (1D) surrogate motions. We evaluated the accuracy of this phantom and performed patient-specific dosimetric QA of surrogate signal-based DTT-IMRT.

## 2. MATERIALS AND METHODS

### 2.A. Construction of a four-axis moving phantom

**FIG. 1.**
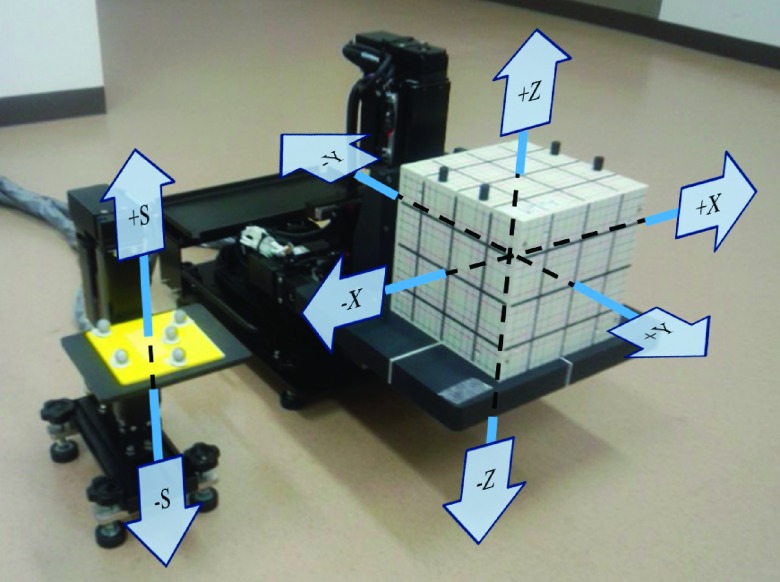
Photograph of the novel four-axis moving phantom. Three axes move along the left–right (*X*), superior-inferior (SI) (*Y*), and anterior–posterior (AP) (*Z*) directions for target motion, and one axis moves along the AP direction for surrogate motion (*S*).

**FIG. 2.**
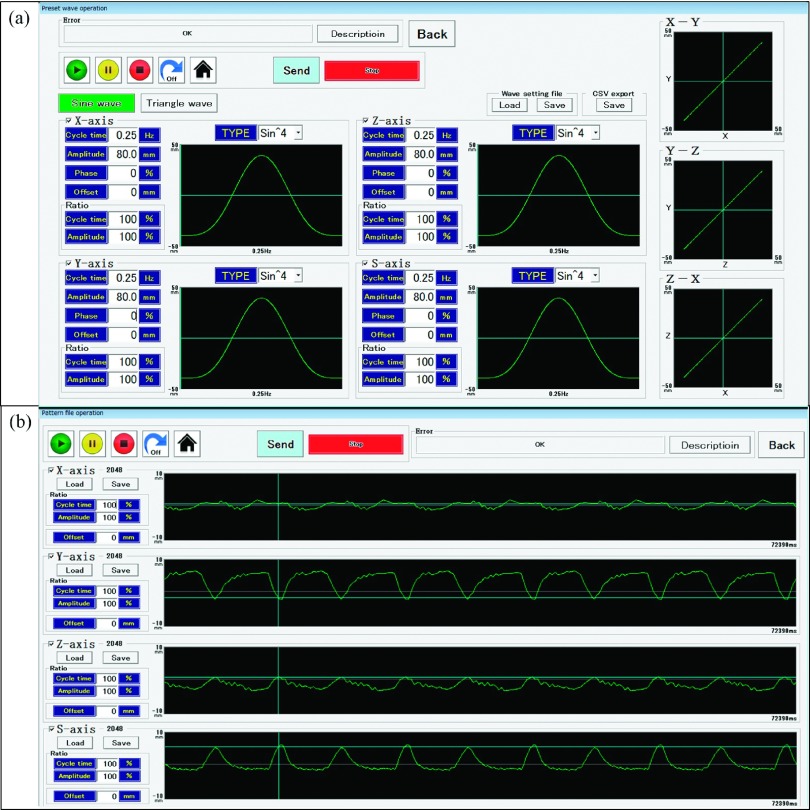
Screen of the operating software for the four-axis moving phantom. The four-axis moving phantom generates (a) preset waves, such as sinusoidal and nth-power-of-sinusoidal patterns or (b) patient respiratory waves using pattern files, in a comma-separated values file format.

The four-axis moving phantom consisted of three orthogonal linear actuators (KR30H06B-120-P; THK Co., Ltd., Tokyo, Japan) that reproduce the 3D target motion with a positional accuracy of 0.02 mm in a motion range of 120 mm with a maximum velocity of 470 mm/s, and a fourth linear actuator (SKR2006A-130-P; THK Co., Ltd.) that reproduces the surrogate motion in the 1D vertical direction with a positional accuracy of 0.02 mm in a motion range of 130 mm with a maximum velocity of 600 mm/s. Figure 1 shows a photograph of the novel four-axis moving phantom. Three axes move along the left–right (LR: *X*), superior-inferior (SI: *Y*), and anterior–posterior (AP: *Z*) directions for target motion, and one axis moves along the AP direction for the surrogate motion (*S*). Dosimetric QA phantoms can be mounted on a driven support base of the three orthogonal linear actuators. The three orthogonal linear actuators for the target motion have a load-bearing capacity of 16 kg in the AP direction and 28 kg in the LR and SI directions. The fourth linear actuator has a load-bearing capacity of 6 kg in the AP direction. The moving phantom was designed to reproduce respiratory motion with a motion range of 100 mm and a maximum velocity of 300 mm/s in each axis. The four axes of the moving phantom are integrated into a system controller that reproduces an arbitrary 3D target and 1D surrogate movements. Figure 2 shows the screen of the operating software for the four-axis moving phantom. The four-axis moving phantom generates preset waves, such as sinusoidal and nth-power-of-sinusoidal patterns, or patient respiratory wave patterns using pattern files, in a comma-separated values file format.

### 2.B. Accuracy verification of the moving phantom

**FIG. 3.**
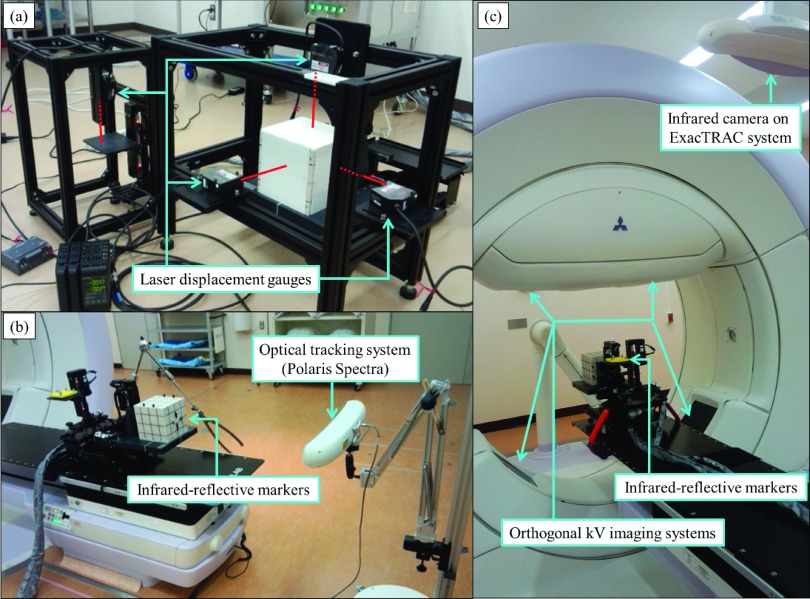
Photographs of the experimental system during verification of the moving phantom for (a) positional accuracy, using the four laser displacement gauges, (b) perpendicularity, using the optical measurement system, and (c) reproducibility of prediction accuracy, using the Vero4DRT.

Figure 3 shows photographs of the experimental system during the accuracy verification process for the moving phantom. The positional accuracy, perpendicularity, and reproducibility of the prediction accuracy of the moving phantom were verified using various independent measurement systems. Each pattern of movement was created with a mounted “I’m*RT* Phantom,” which weighs 6.1 kg (IBA Dosimetry GmbH, Schwarzenbruck, Germany).

First, the positional accuracy of the moving phantom was verified under static conditions with displacements of ±40 mm along each axis. Four laser displacement gauges (LK-G5000; Keyence Corp., Osaka, Japan), with a measurement accuracy of 0.02 mm, were used as an independent verification system for positioning of the phantom. Then, root mean squares (RMSs) of the differences between the measured and commanded phantom positions were evaluated along all axes every 10 ms under moving conditions including regular and irregular motion patterns. Regular motion patterns with four sinusoidal and four fourth-power-of-sinusoidal patterns [peak-to-peak motion range (*H*): 10–80 mm; breathing period (*T*): 4 s] and irregular motion patterns with three patients’ respiratory patterns (*H*: 1.4–2.5 mm in the *X*, 7.7–11.6 mm in the *Y*, 3.1–4.2 mm in the *Z*, and 4.8–14.5 mm in the *S* directions; *T*: 3.9–4.9 s) were used. The absolute velocities (|*V*|) were 5.0–40.0 mm/s in all axes for regular motion patterns and 1.0–1.6 mm/s in the *X*, 2.9–4.7 mm/s in the *Y*, 2.0–2.6 m/s in the *Z*, and 2.4–3.4 mm/s in the *S* directions for irregular motion patterns. |*V*| was calculated by averaging the absolute ratio of the displacement of the phantom according to sampling interval time of 10 ms. The absolute accelerations (|*A*|) were 7.9–81.6 mm/s^2^ in all axes for regular motion patterns and 18.3–33.1 mm/s^2^ in the *X*, 20.2–31.7 mm/s^2^ in the *Y*, 25.8–34.3 mm/s^2^ in the *Z*, and 31.3–43.4 mm/s^2^ in the *S* directions for irregular motion patterns. |*A*| was calculated by averaging the absolute ratio of the variation in the velocity to the sampling interval time of 10 ms.

The perpendicularity of the three axes used for target motion was then verified using the 3D optical position measurement system Polaris Spectra (Northern Digital, Inc., ON, Canada), with a measurement accuracy of 0.3 mm. The angles between each axis of the moving phantom were calculated from the 3D positions of infrared-reflective markers attached to the surface of the I’m*RT* Phantom.

The reproducibility of the prediction accuracy was assessed for the respiratory motions of 20 patients (ten lung, five liver, and five pancreatic cancer patients) who underwent DTT with the Vero4DRT. The Vero4DRT created log files that contained the displacement of the surrogate signals, and the detected and predicted target positions during the 20 s prediction modeling period in DTT. Displacement of the surrogate signals was measured by the infrared camera of the ExacTRAC system (Brainlab AG) with an acquisition interval of 16.7 ms. The detected target positions were defined as the target positions calculated from the detected positions of the implanted fiducial markers around the tumor in the patient or the embedded fiducial markers in the I’m*RT* Phantom using the orthogonal kV x-ray imaging subsystem, with a detection accuracy of 0.2 mm. The corresponding predicted target positions were calculated from a prediction model, expressed as a quadratic equation involving two variables of the positions and velocities of the surrogate signals.^11^ The positions of the surrogate markers were predicted linearly from past motions to compensate for the system delay in DTT of 50 ms.^13^ The prediction errors, defined as mean + two standard deviations (*μ* + 2 SDs) of the absolute difference between the predicted and detected target positions ($E_{\text{predict}}^{\mu +2\;\text{SD}}$) with a sampling interval of 80 ms, from the original respiratory motions were compared with those from the respiratory motions reproduced by the moving phantom. The characteristics of the reproduced respiratory motions, including *H*, |*V*|, and *T* of the target and surrogate motions, and the absolute correlation between the target and surrogate motions (|*R*|), were also calculated from the detected positions in the log files and then compared with the original values. *H* was calculated by averaging the motion range of respiration in each breathing period. *T* was calculated by averaging the duration of each end-inhalation or each end-exhalation.

### 2.C. Patient-specific dosimetric QA for DTT-IMRT

We performed patient-specific dosimetric QA using real respiratory patterns from ten pancreatic cancer patients who underwent DTT-IMRT using the Vero4DRT. Prior to treatment, a dry-run treatment session of DTT was performed to acquire patients’ 3D target and 1D surrogate motions, and to create the prediction model of the moving target position.^11,12^ During DTT treatment, intrafractional internal/external correlation changes due to baseline drift of the respiratory motion reduced the prediction accuracy.^18,19^ Thus, we acquired two prediction models at 10 min intervals in the dry-run treatment session to assume the actual DTT treatment time. The less-accurate prediction model was used for patient-specific dosimetric QAs of DTT-IMRT. Then the four-axis moving phantom mounting I’m*RT* Phantom was actuated, based on the patients’ respiration from log files in DTT. *H* was 2.7 ± 1.4 mm in the *X*, 10.2 ± 1.7 mm in the *Y*, 4.0 ± 1.0 mm in the *Z*, and 11.4 ± 1.8 mm in the 3D directions for the target, and 7.4 ± 2.7 mm in the *S* direction for the surrogate. The $E_{\text{predict}}^{\mu +2\;\text{SD}}$ was 0.7 ± 0.3 mm in the *X*, 0.8 ± 0.2 mm in the *Y*, 0.8 ± 0.3 mm in the *Z*, and 1.4 ± 0.3 mm in the 3D directions. Six-field step-and-shoot IMRT plans were generated using iPlan RT Dose (ver. 4.5; Brainlab AG). 4DCT images were acquired in axial cine mode using a 16-slice CT scanner (LightSpeed RT16; GE Healthcare, Little Chalfont, UK) and a real-time positioning management system (Varian Medical Systems, Palo Alto, USA). The ten phase images were registered to the mid-ventilation phase image based on the centroid of a Visicoil (IBA Dosimetry GmbH) implanted around the tumor. Gross tumor volumes and clinical target volumes (CTVs) and organs at risk (OARs) were delineated on ten respiratory phase images of 4DCT. Then an internal target volume (ITV) for tracking was defined as a composite of the ten CTVs registered, based on the marker centroid. The internal volumes of OARs for tracking were also generated. Because the phase images were registered based on the fiducial marker centroid, the ITV for tracking and internal volumes of OARs for tracking were supposed to compensate for target and OAR deformations and their positional uncertainty from the fiducial marker during respiration. A planning target volume (PTV) margin of 5 mm or more was added to the ITV, depending on intra and interfractional tracking uncertainties.^11,12^ Planning OAR volumes (PRVs) were also generated by adding margins of 3–5 mm for the interfractional uncertainties to internal volumes of OARs. The dose calculation was performed on the mid-ventilation phase image using the x-ray Voxel Monte Carlo (XVMC) algorithm. The spatial resolution and variance of dose calculation were set to 2 mm and 2%, respectively. The prescribed dose of 45–48 Gy in 15 fractions covered 95% of the PTV excluding PRVs. The size of the PTV was 190.7 ± 50.5 cm^3^. The IMRT plans were then delivered to the I’m*RT* Phantom under three scenarios: static, moving, and tracking conditions. During beam delivery, the Vero4DRT tracked the moving target, reproducing the prediction error under the tracking conditions. Meanwhile, the mean position of the moving target was set to the isocenter, and doses were delivered without tracking under moving conditions. The absolute dose distributions delivered to the Gafchromic EBT3 films (Ashland, Inc., Wayne, NJ, USA) with or without tracking, inserted in the coronal and sagittal planes at the planned isocenter of the I’m*RT* Phantom, were compared with those under static conditions without dose normalization. In total, 60 films were scanned in transmission mode in the same orientation using a resolution of 72 dpi in the 16-bit red-channel color scale with a constant 24-h post-exposure period. Four pinholes were made in each film to identify the planned isocenter. All films were analyzed using commercially available radiation dosimetry software (DD system, ver. 9.4; R’Tech, Inc., Tokyo, Japan). After the density-to-dose conversion, translational and rotational corrections were performed; then, corresponding dose data were exported to in-house software developed with Visual Basic for Applications to analyze the dose difference and *γ* with a global difference approach. The passing rates for *γ* with criteria of 3%/1 mm (*γ*_3%/1 mm_) and 3%/3 mm (*γ*_3%/3 mm_), and dose differences with criteria of 3% (DD_3%_) and 5% (DD_5%_), were calculated as the patient-specific dosimetric QA with a 10% planar dose maximum threshold under the static condition.

## 3. RESULTS

### 3.A. Accuracy verification of the moving phantom

**FIG. 4.**
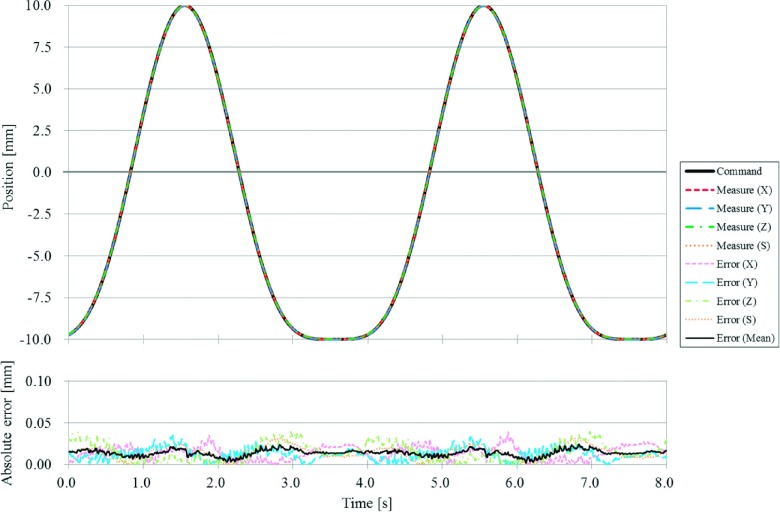
Representative results of variations in the measured and commanded positions of the moving phantom for a fourth-power-of-sinusoidal pattern with a peak-to-peak motion range (*H*) of 20 mm and a breathing period (*T*) of 4 s.

**FIG. 5.**
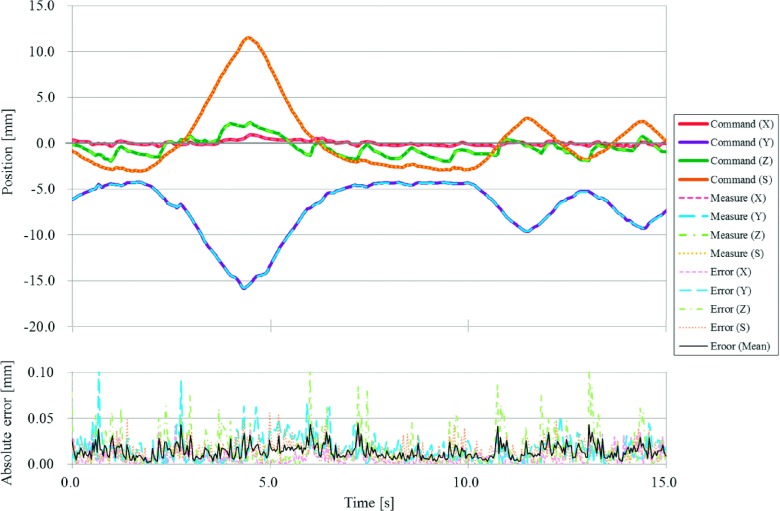
Representative results of variation in the measured and commanded positions of the moving phantom for an irregular respiratory pattern with a *H* of 0.7 ± 0.4 mm in the *X*, 6.6 ± 3.4 mm in the *Y*, 2.8 ± 0.8 mm in the *Z*, and 7.7 ± 4.7 mm in the *S* directions, and a *T* of 5.2 ± 2.2 s.

**FIG. 6.**
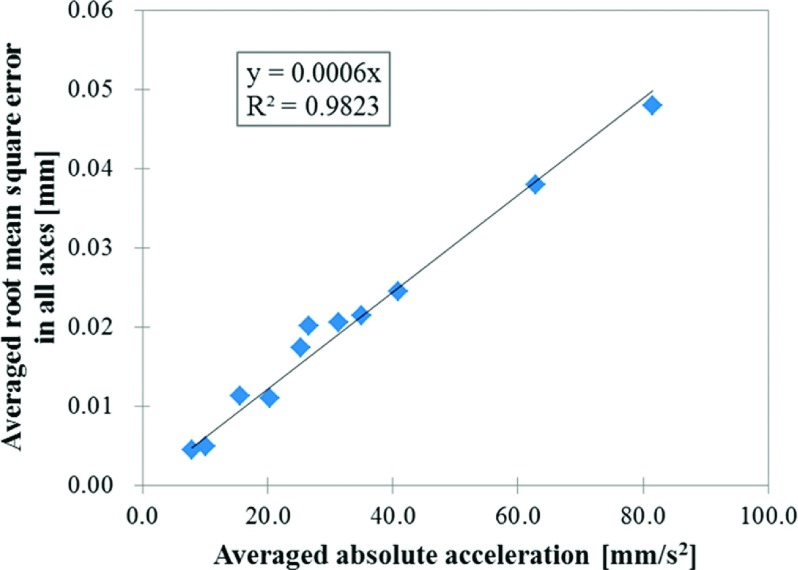
Relationship between the variation in averaged absolute acceleration and averaged root mean square error in all axes for regular and irregular motion patterns.

The positional accuracy of the moving phantom under static conditions was up to 0.03 mm in the *X*, 0.05 mm in the *Y*, 0.03 mm in the *Z*, and 0.03 mm in the *S* directions. The RMSs of the positional error under the moving conditions were within 0.05 mm in all directions. Figure 4 shows representative results of variations in the measured and commanded positions of the moving phantom for a fourth-power-of-sinusoidal pattern with a *H* of 20 mm and a *T* of 4 s. The averaged RMS of the positional error for this regular motion pattern was 0.01 mm. Figure 5 shows representative results of variation in the measured and commanded positions of the moving phantom for an irregular respiratory pattern with a *H* of 0.7 ± 0.4 mm in the *X*, 6.6 ± 3.4 mm in the *Y*, 2.8 ± 0.8 mm in the *Z*, and 7.7 ± 4.7 mm in the *S* directions, and a *T* of 5.2 ± 2.2 s. The RMS of the positional error for this irregular motion pattern was 0.01 mm in the *X*, 0.02 mm in the *Y*, 0.03 mm in the *Z*, and 0.02 mm in the *S* directions, and 0.02 mm on average. A strong positive correlation was found between |*A*| and the averaged RMS of the positional error in all directions (|*R*| = 0.99; Fig. 6). Furthermore, the perpendicularity of the three axes for the target motion was within 0.2° from 90° along all axes. A summary of the reproducibility of the moving phantom for the respiratory motion of 20 patients is shown in Table I. The *μ* + 2 SDs of differences between the original respiratory motions and the ones reproduced by the moving phantom were within 0.8 mm for *H*, 0.5 s for *T*, 0.7 mm/s for |*V*|, 0.09 for |*R*|, and 0.4 mm for $E_{\text{predict}}^{\mu +2\;\text{SD}}$ in all directions.

**TABLE I.** Reproducibility of the moving phantom for 20 patient respiratory motions.

*μ* ± SD		Original motion	Reproduced motion	Difference in each motion
*H* (mm)	*X*	1.9 ± 1.3	1.9 ± 1.3	−0.1 ± 0.1
	*Y*	11.1 ± 4.7	11.0 ± 4.8	0.0 ± 0.4
	*Z*	3.3 ± 1.0	3.1 ± 1.0	−0.2 ± 0.2
	*S*	6.9 ± 3.1	6.8 ± 3.1	0.0 ± 0.3
*T* (s)	*X*	4.0 ± 0.9	4.1 ± 0.9	0.1 ± 0.2
	*Y*	4.0 ± 0.9	4.1 ± 0.9	0.1 ± 0.2
	*Z*	4.0 ± 0.9	4.1 ± 0.8	0.0 ± 0.2
	*S*	4.0 ± 0.9	4.1 ± 0.9	0.1 ± 0.2
|*V*| (mm/s)	*X*	1.3 ± 0.6	1.2 ± 0.5	−0.1 ± 0.1
	*Y*	5.6 ± 1.9	5.6 ± 2.0	0.0 ± 0.2
	*Z*	2.1 ± 0.8	1.9 ± 0.6	−0.2 ± 0.3
	*S*	3.5 ± 1.2	3.6 ± 1.2	0.1 ± 0.2
|*R*|	*X*	0.76 ± 0.23	0.76 ± 0.23	0.00 ± 0.04
	*Y*	0.98 ± 0.03	0.98 ± 0.03	0.00 ± 0.00
	*Z*	0.80 ± 0.23	0.79 ± 0.25	−0.01 ± 0.04
$E_{\text{predict}}^{\mu +2\;\text{SD}}$ (mm)	*X*	0.6 ± 0.4	0.5 ± 0.4	−0.1 ± 0.1
	*Y*	1.3 ± 0.5	1.1 ± 0.5	−0.1 ± 0.2
	*Z*	0.8 ± 0.6	0.7 ± 0.5	−0.1 ± 0.1

Note: Abbreviations: *μ*—mean; SD—standard deviation; *H*—peak-to-peak motion range; *T*—breathing period; |*V*|—average absolute velocity; |*R*|—absolute correlation between target and surrogate motions; $E_{\text{predict}}^{\mu +2\;\text{SD}}$, *μ* + 2 SD of the absolute error of the prediction model; *X*, *Y*, and *Z*, the left–right, superior-inferior, and anterior–posterior directions of target motion, respectively; *S*, the anterior–posterior direction for surrogate motion.

### 3.B. Patient-specific dosimetric QA for DTT-IMRT

**FIG. 7.**
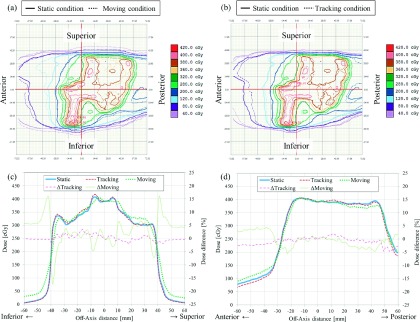
Representative results of patient-specific dosimetric QA in the sagittal plane. The isodose distribution under the static condition (solid line) was compared with that under the (a) moving and (b) tracking conditions (dashed lines). The dose profile under the static, moving, and tracking conditions (solid, dashed, and dotted lines, respectively) is drawn along the (c) SI and (d) AP directions.

**FIG. 8.**
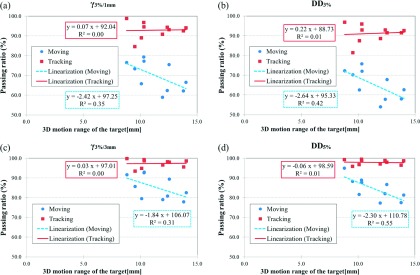
Relationship between the 3D motion range of the target and the dosimetric accuracy of *γ* with criteria of (a) 3%/1 mm (*γ*_3%/1 mm_) and (b) 3%/3 mm (*γ*_3%/3 mm_), and dose differences with criteria of (c) 3% (DD_3%_) and (d) 5% (DD_5%_).

**FIG. 9.**
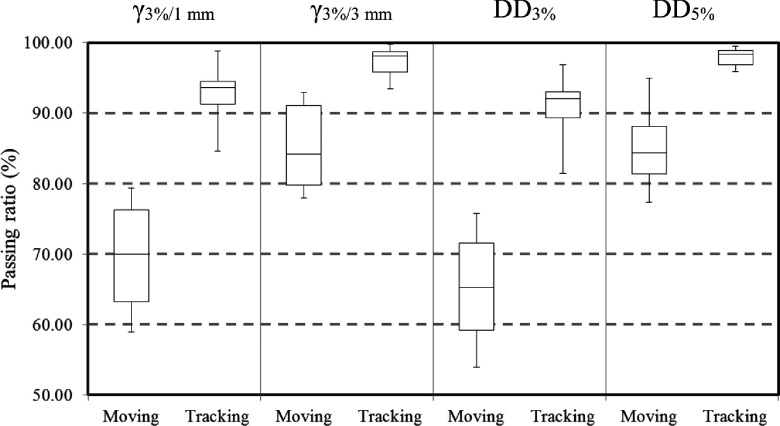
Box plot for the summary of measurement-based patient-specific dosimetric QA. The top, middle, and bottom lines of the box are the first, second, and third quartiles. The ends of the whiskers are maximum and minimum values.

The absolute dose distribution under the static condition was not consistent with that under the moving condition, but was consistent with that under the tracking condition. Figure 7 shows a representative result for patient-specific dosimetric QA. Although the isodose distribution under the tracking condition was consistent with the static condition, a blurring effect was observed under the moving condition. Dose differences over 5% were observed in the steep gradient region under the moving condition. The *γ*_3%/1 mm_, *γ*_3%/3 mm_, DD_3%_, and DD_5%_ under conditions of moving/tracking were 69.8% ± 7.4%/92.9% ± 4.0%, 85.2% ± 6.0%/97.3% ± 2.1%, 65.3% ± 7.4%/91.2% ± 4.5%, and 84.6% ± 5.6%/97.9% ± 1.3%, respectively. The dosimetric accuracy was improved significantly by tracking for all evaluated dosimetric parameters (*p* < 0.0001). Figure 8 shows relationships between the 3D motion range of the target and the dosimetric accuracy. Although the dosimetric accuracy of *γ*_3%/1 mm_, *γ*_3%/3 mm_, DD_3%_, and DD_5%_ without tracking showed a significant negative correlation with the 3D motion range of the target (*r* = − 0.59, *p* = 0.036; *r* = − 0.55, *p* = 0.048; *r* = − 0.65, *p* = 0.021; and *r* = − 0.74, *p* = 0.007, respectively), there was no significant correlation with tracking (*r* = 0.03, *p* = 0.464; *r* = 0.02, *p* = 0.474; *r* = 0.09, *p* = 0.404; and *r* = − 0.08, *p* = 0.410, respectively). As the 3D motion range of the target increased, the dosimetric accuracy without tracking decreased significantly but that with tracking remained unchanged. The averaged mean dose difference and averaged mean values of *γ*_3%/1 mm_ and *γ*_3%/3 mm_ under conditions of moving/tracking were 0.2% ± 0.8%/−0.1% ± 0.5%, 0.9 ± 0.2/0.4 ± 0.1, and 0.6 ± 0.1/0.3 ± 0.1, respectively. Although there was no significant difference in mean dose with or without tracking (*p* = 0.196), the mean values of *γ*_3%/1 mm_ and *γ*_3%/3 mm_ did show a significant difference (*p* < 0.0001). Figure 9 shows a box plot summarizing measurement-based patient-specific dosimetric QA. The top, middle, and bottom lines of the box are the first, second, and third quartiles. The ends of the whiskers represent maximum and minimum values. The dosimetric accuracy was improved with tracking relative to that without tracking.

## 4. DISCUSSION

DTT-IMRT is currently available in clinical practice by combining IMRT with DTT. Dosimetric uncertainties, including IMRT- and DTT-related uncertainties, should be verified under realistic conditions. We developed a four-axis moving phantom that reproduced patient 3D tumor and 1D surrogate motions to realize truly patient-specific dosimetric QA for surrogate signal-based DTT-IMRT. Additionally, we assessed the accuracy of the moving phantom, and then conducted patient-specific QA for pancreatic cancer patients who underwent surrogate signal-based DTT-IMRT.

Many moving phantoms have been designed for the QA of motion management. QUASAR (Modus Medical Devices, Inc., London, ON, Canada), CIRS (Computerized Imaging Reference Systems, Inc., Norfolk, VA, USA), and HexaMotion (ScandiDos AB, Uppsala, Sweden) are available commercially. However, the surrogate axis of the QUASAR phantom works with dependent motion of the target axis. The CIRS phantom has a semi-independent surrogate axis, which generates a phase shift between target and surrogate motions. The HexaMotion, a five-dimensional moving platform in combination with Delta4 (ScandiDos AB), however, has no axis for surrogate motions; thus, it can be used in direct, but not indirect, tracking.^2,9^ In addition, noncommercially available moving phantoms have been developed.^27–32^ Although some phantoms can reproduce 3D target motions, they have no axis for surrogate motions. Zhou *et al.* and Malinowski *et al.* developed four-axis moving phantoms, which were able to reproduce the movements of both the target and surrogate.^29,30^ A major challenge in designing a moving phantom is to avoid radiopaque materials that will absorb an x-ray beam. The three axes for the 3D target motion in the moving phantom developed by Zhou *et al.*^29^ consisted of a unique geometry, two linear slides, and one vertically mounted linear piston actuator, for the CyberKnife tracking system, which uses two orthogonal kV x-ray beams to detect internal fiducials. Thus, only radiolucent materials may be present in the paths of these kV x-ray beams; however, radiopaque materials are present in the path of the MV x-ray beam from the posterior direction. The three axes for 3D target motion in the moving phantom developed by Malinowski *et al.*^30^ consisted of three linear motion slides with rotary stepper motors (BiSlide, Velmex, Inc, Bloomfield, NY). The purpose of this moving phantom was patient-specific, end-to-end radiation therapy QA, especially for lung tumors. The weight-bearing capacity of this phantom affected the maximum speed of its movement. They used a tumor phantom, composed of an acrylic sphere 1.5 cm in diameter, encased in solid water, affixed to the support arm of the 3D stage to confirm the accuracy of 4D CT. If the same dosimetric phantom of 6.1 kg were used for the patient QA of DTT-IMRT for pancreas tumor, the speed of the motion phantom for lifting would be limited to about 7.5 revolution/s (0.5–15 mm/s). Furthermore, the accuracies of the phantoms were verified by mounting only lightweight phantoms. In the present study, the *μ* + 2 SD of the PTV in DTT-IMRT was 291.6 cm^3^, which corresponds to a sphere of ∼82 mm in diameter. To estimate the dose distribution of such a large target volume and the surrounding normal tissues, a relatively large dosimetric phantom is required. Because a large dosimetric phantom is heavy, the accuracy of the moving phantom needs to be confirmed with a mounted dosimetric phantom. Haas *et al.* developed the MAESTRO thorax phantom, which has moving ribs and moving insertion of a target within the lung.^31^ This phantom is useful for performing dosimetric verification of surrogate signal-based DTT in lung cancer patients. An inhomogeneous phantom reproducing patient geometry will enable verification of the dose distribution with inhomogeneity correction, while a geometric error in the tissue may cause over or underestimation of the dose distribution. Jung *et al.* developed individualized lung phantoms that closely mimicked the lung anatomy of actual patients using 3D printing technology.^32^ The individualized lung inserts and QUASAR respiratory motion phantom were combined to confirm the accuracy of the direct tracking system.

The four-axis moving phantom developed here can mount a dosimetric QA phantom, such as the I’m*RT* phantom (which may contain homogeneous and inhomogeneous inserts), and independently reproduce the patient’s respiration in the 3D target and 1D surrogate motions with a motion range of 100 mm. The newly developed moving phantom can cover respiratory motion of up to 34 mm in the SI, 24 mm in the AP, and 16 mm in the LR directions (values taken from AAPM TG76).^2^

The positional accuracy of our novel moving phantom was within 0.05 mm under static and moving conditions in all axes. During the patient’s irregular respiratory pattern, instantaneous positional error was observed (Fig. 5). This was caused in part by the noise of the respiratory motion acquired with a measurement accuracy of 0.2 mm for the internal target motion and 0.3 mm for the external surrogate motion. While high velocity increased the acceleration and inertial force, and led to a greater positional error, the increase in positional error was still negligible even when the dosimetric phantom was mounted (Fig. 6). The perpendicularity of the moving phantom was also found to be within 0.2° of 90° along all axes, which was within the measurement accuracy of the Polaris spectra. Additionally, from the results of the reproducibility tests, motion characteristics and prediction model accuracies of the reproduced respiratory motions were found to be consistent with the original ones, even for irregular respiratory motions.

As shown in this study, the four-axis moving phantom enables patient-specific dosimetric QA to be performed for surrogate signal-based DTT-IMRT. In the present study, dosimetric accuracies for moving and tracking conditions were assessed based on the dose distribution under the static condition directly, to assess the feasibility of tracking QA using the moving phantom and the benefit of the DTT-IMRT. In a clinical setting, patient-specific dosimetric QA should be performed based on the dose distribution calculated with XVMC with a variance of 2%. Thus, dosimetric QA for the calculated dose distribution would include both tracking and dose calculation uncertainties. One of the main characteristics of this phantom is that the uncertainties in the surrogate signal-based DTT-IMRT can be estimated by performing patient-specific dosimetric QA. However, the phantom has a limitation with regard to the reproduction of the deformation of the patient’s external shape. The moving phantom reproduces the target motion by translating the phantom itself. Thus, changes in the source-to-surface distance or depth to the target during beam delivery differ between the patient and phantom, which will lead to dosimetric errors. However, dosimetric variations along the beam axis will appear randomly under moving conditions, and will, in part, be averaged out. Although the peripheral dose to the PTV in our study was decreased under the moving condition, the doses delivered to the center of the PTV were consistent even without dose normalization (Fig. 7). Furthermore, averaged mean dose differences were within 0.2% under moving and tracking conditions and there were no significant differences. Both target motion and residual tracking error caused interplay effects that increased the dose to a partial volume and decreased the dose to a partial volume; however, the total delivered doses under the moving and tracking conditions were consistent with that under the static condition. Thus, there was no systematic error in the present dosimetric verification system or the beam delivery system with or without tracking. The positional tracking error perpendicular to the beam axis causes a dosimetric difference, compared to the static condition (Fig. 7). The positional tracking error causes blurring on the dose profiles of each segment in the IMRT, which was increased as target motion increased (Fig. 8). In the IMRT treatment of pancreatic tumor, dose differences above 5% are clinically unacceptable in the dose gradient region inside the PTV, which is the overlapping region of the PTV and organs surrounding the pancreatic tumor that are at risk, such as the duodenum, stomach, and bowels (Fig. 7). Colvill *et al.* conducted a multi-institutional dosimetric comparison study, of real-time adaptive and non-adaptive radiotherapy.^33^ The respiratory motion resulted in much higher *γ*-fail rates without motion adaptation, while *γ*-fail rates were comparable for conventional IMRT under the static condition.^33,34^ The dosimetric results of the present study were consistent with that study. According to the ESTRO guidelines, a passing ratio of *γ* < 90% and a mean value of *γ* > 0.6 are not acceptable, which were achieved with tracking, but not achieved without tracking.^35^ The passing ratio of *γ* and mean value of *γ* were significantly improved with tracking, which means that dosimetric errors caused by respiratory motion were compensated for in all parts of the dose distribution. Overall, it was found that the dosimetric accuracy was sufficiently high in the surrogate signal-based DTT-IMRT with the Vero4DRT (Figs. 8 and 9).

## 5. CONCLUSIONS

In this study, we developed a four-axis moving phantom that was able to reproduce patient 3D tumor and 1D surrogate motions. The four-axis moving phantom had sufficient accuracy and weight-bearing capacity with respect to reproduction of patient respiratory motions mounting the dosimetric phantom. Thus, patient-specific dosimetric QA of surrogate signal-based DTT-IMRT can be performed under realistic conditions using the moving phantom.

## References

[1] L. Veldeman, I. Madani, F. Hulstaert, G. De Meerleer, M. Mareel, and W. De Neve, “Evidence behind use of intensity-modulated radiotherapy: A systematic review of comparative clinical studies,” Lancet Oncol. 9, 367–375 (2010). 10.1016/S1470-2045(08)70098-6 18374290

[2] P. J. Keall, G. S. Mageras, R. S. Balter, R. S. Emery, K. M. Forster, S. B. Jiang, J. M. Kapatoes, D. A. Low, M. J. Murphy, B. R. Murray, C. R. Ramsey, M. B. Van Herk, S. S. Vedam, J. W. Wong, and E. Yorke, “The management of respiratory motion in radiation oncology: Report of AAPM Radiation Therapy Committee Task Group No. 76,” Med. Phys. 33, 3874–3900 (2006). 10.1118/1.2349696 17089851

[3] T. Bortfeld, S. B. Jiang, and E. Rietzel, “Effects of motion on the total dose distribution,” Semin. Radiat. Oncol. 14, 41–51 (2004). 10.1053/j.semradonc.2003.10.011 14752732

[4] L. E. Court, M. Wagar, D. Ionascu, R. Berbeco, and L. Chin, “Management of the interplay effect when using dynamic MLC sequences to treat moving targets,” Med. Phys. 35, 1926–1931 (2008). 10.1118/1.2896083 18561668

[5] H. Chen, A. Wu, E. D. Brandner, D. E. Heron, M. S. Huq, N. J. Yue, and W. C. Chen, “Dosimetric evaluations of the interplay effect in respiratory-gated intensity-modulated radiation therapy,” Med. Phys. 36, 893–903 (2009). 10.1118/1.3070542 19378749

[6] B. J. Waghorn, A. P. Shah, J. M. Rineer, K. M. Langen, and S. L. Meeks, “A margin-based analysis of the dosimetric impact of motion on step-and-shoot IMRT lung plans,” Radiat. Oncol. 9, 46 (8pp.) (2014). 10.1186/1748-717X-9-46 24499602 PMC3922402

[7] Y. Matsuo, K. Shibuya, M. Nakamura, M. Narabayashi, K. Sakanaka, N. Ueki, K. Miyagi, Y. Norihisa, T. Mizowaki, Y. Nagata, and M. Hiraoka, “Dose-volume metrics associated with radiation pneumonitis after stereotactic body radiation therapy for lung cancer,” Int. J. Radiat. Oncol., Biol., Phys. 83, e545–e549 (2012). 10.1016/j.ijrobp.2012.01.018 22436782

[8] A. Nakamura, K. Shibuya, Y. Matsuo, M. Nakamura, T. Shiinoki, T. Mizowaki, and M. Hiraoka, “Analysis of dosimetric parameters associated with acute gastrointestinal toxicity and upper gastrointestinal bleeding in locally advanced pancreatic cancer patients treated with gemcitabine-based concurrent chemoradiotherapy,” Int. J. Radiat. Oncol., Biol., Phys. 84, 369–375 (2012). 10.1016/j.ijrobp.2011.12.026 22381898

[9] S. Dieterich, K. Cleary, W. D’Souza, M. Murphy, K. H. Wong, and P. Keall, “Locating and targeting moving tumors with radiation beams,” Med. Phys. 35, 5684–5694 (2008). 10.1118/1.3020593 19175125

[10] S. S. Korreman, T. Juhler-Nottrup, and A. L. Boyer, “Respiratory gated beam delivery cannot facilitate margin reduction, unless combined with respiratory correlated image guidance,” Radiother. Oncol. 86, 61–68 (2008). 10.1016/j.radonc.2007.10.038 18039549

[11] N. Mukumoto, M. Nakamura, M. Yamada, K. Takahashi, H. Tanabe, S. Yano, Y. Miyabe, N. Ueki, S. Kaneko, Y. Matsuo, T. Mizowaki, A. Sawada, M. Kokubo, and M. Hiraoka, “Intrafractional tracking accuracy in infrared marker-based hybrid dynamic tumour-tracking irradiation with a gimballed linac,” Radiother. Oncol 111, 301–305 (2014). 10.1016/j.radonc.2014.02.018 24746563

[12] Y. Matsuo, N. Ueki, K. Takayama, M. Nakamura, Y. Miyabe, Y. Ishihara, N. Mukumoto, S. Yano, H. Tanabe, S. Kaneko, T. Mizowaki, H. Monzen, A. Sawada, M. Kokubo, and M. Hiraoka, “Evaluation of dynamic tumour tracking radiotherapy with real-time monitoring for lung tumours using a gimbal mounted linac,” Radiother. Oncol. 112, 360–364 (2014). 10.1016/j.radonc.2014.08.003 25154320

[13] T. Depuydt, D. Verellen, O. Haas, T. Gevaert, N. Linthout, M. Duchateau, K. Tournel, T. Reynders, K. Leysen, M. Hoogeman, G. Storme, and M. De Ridder, “Geometric accuracy of a novel gimbals-based radiation therapy tumor tracking system,” Radiother. Oncol. 98, 365–372 (2011). 10.1016/j.radonc.2011.01.015 21316786

[14] T. Depuydt, K. Poels, D. Verellen, B. Engels, C. Collen, C. Haverbeke, T. Gevaert, N. Buls, G. Van Gompel, T. Reynders, M. Duchateau, K. Tournel, M. Boussaer, F. Steenbeke, F. Vandenbroucke, and M. De Ridder, “Initial assessment of tumor tracking with a gimbaled linac system in clinical circumstances: A patient simulation study,” Radiother. Oncol. 106, 236–240 (2013). 10.1016/j.radonc.2012.12.015 23398905

[15] M. Hoogeman, J. B. Prevost, J. Nuyttens, J. Pöll, P. Levendag, and B. Heijmen, “Clinical accuracy of the respiratory tumor tracking system of the CyberKnife: Assessment by analysis of log files,” Int. J. Radiat. Oncol., Biol., Phys. 74, 297–303 (2009). 10.1016/j.ijrobp.2008.12.041 19362249

[16] H. Bahig, M. P. Campeau, T. Vu, R. Doucet, D. Béliveau Nadeau, B. Fortin, D. Roberge, L. Lambert, J. F. Carrier, and E. Filion, “Predictive parameters of CyberKnife fiducial-less (XSight lung) applicability for treatment of early non-small cell lung cancer: A single-center experience,” Int. J. Radiat. Oncol., Biol., Phys. 87, 583–589 (2013). 10.1016/j.ijrobp.2013.06.2048 23953636

[17] P. J. Keall, E. Colvill, R. O’Brien, J. A. Ng, P. R. Poulsen, T. Eade, A. Kneebone, and J. T. Booth, “The first clinical implementation of electromagnetic transponder-guided MLC tracking,” Med. Phys. 41, 020702 (5pp.) (2014). 10.1118/1.4862509 24506591 PMC3977852

[18] K. Malinowski, T. J. McAvoy, R. George, S. Dietrich, and W. D. D’Souza, “Incidence of changes in respiration-induced tumor motion and its relationship with respiratory surrogates during individual treatment fractions,” Int. J. Radiat. Oncol., Biol., Phys. 82, 1665–1673 (2012). 10.1016/j.ijrobp.2011.02.048 21498009

[19] M. Akimoto, M. Nakamura, N. Mukumoto, H. Tanabe, M. Yamada, Y. Matsuo, H. Monzen, T. Mizowaki, M. Kokubo, and M. Hiraoka, “Predictive uncertainty in infrared marker-based dynamic tumor tracking with Vero4DRT,” Med. Phys. 40, 091705 (8pp.) (2013). 10.1118/1.4817236 24007138

[20] I. Buzurovic, Y. Yu, M. Werner-Wasik, T. Biswas, P. R. Anne, A. P. Dicker, and T. K. Podder, “Implementation and experimental results of 4D tumor tracking using robotic couch,” Med. Phys. 39, 6957–6967 (2012). 10.1118/1.4758064 23127089 PMC3494731

[21] I. Buzurovic, K. Huang, Y. Yu, and T. K. Podder, “A robotic approach to 4D real-time tumor tracking for radiotherapy,” Phys. Med. Biol. 56, 1299–1318 (2011). 10.1088/0031-9155/56/5/005 21285488

[22] I. Buzurovic, Y. Yu, and T. K. Podder, “Active tracking and dynamic dose delivery for robotic couch in radiation therapy,” in *Annual International Conference of the IEEE Engineering in Medicine and Biology Society* (IEEE, New York, 2011), pp. 2156–2159. 10.1109/IEMBS.2011.6090404 22254765

[23] A. Fassi, J. Schaerer, M. Fernandes, M. Riboldi, D. Sarrut, and G. Baroni, “Tumor tracking method based on a deformable 4D CT breathing motion model driven by an external surface surrogate,” Int. J. Radiat. Oncol., Biol., Phys. 88, 182–188 (2014). 10.1016/j.ijrobp.2013.09.026 24331665

[24] S. Dhou, M. Hurwitz, P. Mishra, W. Cai, J. Rottmann, R. Li, C. Williams, M. Wagar, R. Berbeco, D. Ionascu, and J. H. Lewis, “3D fluoroscopic image estimation using patient-specific 4DCBCT-based motion models,” Phys. Med. Biol. 60, 3807–3824 (2015). 10.1088/0031-9155/60/9/3807 25905722 PMC4432909

[25] M. Nakamura, K. Shibuya, A. Nakamura, T. Shiinoki, Y. Matsuo, M. Nakata, A. Sawada, T. Mizowaki, and M. Hiraoka, “Interfractional dose variations in intensity-modulated radiotherapy with breath-hold for pancreatic cancer,” Int. J. Radiat. Oncol., Biol., Phys. 82, 1619–1626 (2012). 10.1016/j.ijrobp.2011.01.050 21477941

[26] J. Boda-Heggemann, S. Mai, J. Fleckenstein, K. Siebenlist, A. Simeonova, M. Ehmann, V. Steil, F. Wenz, F. Lohr, and F. Stieler, “Flattening-filter-free intensity modulated breath-hold image-guided SABR (stereotactic ABlative radiotherapy) can be applied in a 15-min treatment slot,” Radiother. Oncol. 109, 505–509 (2013). 10.1016/j.radonc.2013.09.014 24128805

[27] H. Nakayama, T. Mizowaki, Y. Narita, N. Kawada, K. Takahashi, K. Mihara, and M. Hiraoka, “Development of a three-dimensionally movable phantom system for dosimetric verifications,” Med. Phys. 35, 1643–1650 (2008). 10.1118/1.2897971 18561639

[28] A. H. Belcher, X. Liu, Z. Grelewicz, E. Pearson, and R. D. Wiersma, “Development of a 6DOF robotic motion phantom for radiation therapy,” Med. Phys. 41, 121704 (7pp.) (2014). 10.1118/1.4900828 25471951 PMC4235649

[29] T. Zhou, J. Tang, S. Dieterich, and K. Cleary, “A robotic 3-D motion simulator for enhanced accuracy in CyberKnife stereotactic radiosurgery,” Comput. Assist. Radiol. Surg. 1268, 323–328 (2004). 10.1016/j.ics.2004.03.296

[30] K. Malinowski, C. Noel, W. Lu, K. Lechleitera, J. Hubenschmidta, D. Lowa, and P. Parikh, “Development of the 4D Phantom for patient-specific, end-to-end radiation therapy QA,” Proc. SPIE 6510, 65100E (2007). 10.1117/12.713841

[31] O. C. Haas, J. A. Mills, I. Land, P. Mulholl, P. Menary, R. Crichton, A. Wilson, J. Sage, M. Anna, and T. Depuydt, “IGRT/ART phantom with programmable independent rib cage and tumor motion,” Med. Phys. 41, 022106 (6pp.) (2014). 10.1118/1.4860662 24506638

[32] J. Jung, S. Y. Song, S. M. Yoon, J. Kwak, K. Yoon, W. Choi, S. Y. Jeong, E. K. Choi, and B. Cho, “Verification of accuracy of CyberKnife tumor-tracking radiation therapy using patient-specific lung phantom,” Int. J. Radiat. Oncol., Biol., Phys. 95, 745–753 (2015). 10.1016/j.ijrobp.2015.02.055 25936598

[33] E. Colvill, J. Booth, S. Nill, M. Fast, J. Bedford, U. Oelfke, M. Nakamura, P. Poulsen, E. Worm, R. Hansen, T. Ravkilde, J. Scherman Rydhög, T. Pommer, P. Munck Af Rosenschöld, S. Lang, M. Guckenberger, C. Groh, C. Herrmann, D. Verellen, K. Poels, L. Wang, M. Hadsell, T. Sothmann, O. Blanck, and P. Keall, “A dosimetric comparison of real-time adaptive and non-adaptive radiotherapy: A multi-institutional study encompassing robotic, gimbaled, multileaf collimator and couch tracking,” Radiother. Oncol. 119, 159–165 (2016). 10.1016/j.radonc.2016.03.006 27016171 PMC4854175

[34] G. A. Ezzell, J. W. Burmeister, N. Dogan, T. J. Losasso, J. G. Mechalakos, D. Mihailidis, A. Molineu, J. R. Palta, C. R. Ramsey, and B. J. Salter, “IMRT commissioning: Multiple institution planning and dosimetry comparisons, a report from AAPM Task Group 119,” Med. Phys. 36, 5359–5373 (2009). 10.1118/1.3238104 19994544

[35] M. Alber, S. Broggi, C. D. Wagter, I. Eichwurzel, P. Engström, C. Fiorino, D. Georg, G. Hartmann, T. Knöös, A. Leal, H. Marijnissen, B. Mijnheer, M. Paiusco, F. Sánchez-Doblado, R. Schmidt, M. Tomsej, and H. Welleweerd, “Guidelines for the verification of IMRT,” in *ESTRO Booklet No. 9*, edited by Mijheer B. and Georg D. (ESTRO, Brussels, Belgium, 2008).

